# Granulomatous Lymphocytic Interstitial Lung Disease (GLILD) in Common Variable Immunodeficiency (CVID): A Multicenter Retrospective Study of Patients From Italian PID Referral Centers

**DOI:** 10.3389/fimmu.2021.627423

**Published:** 2021-03-10

**Authors:** Francesco Cinetto, Riccardo Scarpa, Maria Carrabba, Davide Firinu, Vassilios Lougaris, Helena Buso, Giulia Garzi, Sabrina Gianese, Valentina Soccodato, Alessandra Punziano, Gianluca Lagnese, Giulio Tessarin, Giulia Costanzo, Nicholas Landini, Stefania Vio, Maria Pia Bondioni, Dario Consonni, Carolina Marasco, Stefano Del Giacco, Marcello Rattazzi, Angelo Vacca, Alessandro Plebani, Giovanna Fabio, Giuseppe Spadaro, Carlo Agostini, Isabella Quinti, Cinzia Milito

**Affiliations:** ^1^Department of Medicine—DIMED, University of Padova, Padua, Italy; ^2^Internal Medicine I, Ca' Foncello Hospital, AULSS2 Marca Trevigiana, Treviso, Italy; ^3^Internal Medicine Department, Rare Disease Unit, Fondazione IRCCS Ca' Granda Ospedale Maggiore Policlinico, Milan, Italy; ^4^Department of Medical Sciences and Public Health, University of Cagliari, Monserrato, Italy; ^5^Department of Clinical and Experimental Sciences, Pediatrics Clinic and Institute for Molecular Medicine A. Nocivelli, University of Brescia, Brescia, Italy; ^6^ASST-Spedali Civili di Brescia, Brescia, Italy; ^7^Regional Reference Centre for Primary Immune Deficiencies, Azienda Ospedaliera-Universitaria Policlinico Umberto I, Rome, Italy; ^8^Department of Translational Medical Sciences—Center for Basic and Clinical Immunology Research, University of Naples Federico II, Naples, Italy; ^9^Radiology Unit, Ca' Foncello Hospital, AULSS2 Marca Trevigiana, Treviso, Italy; ^10^Radiology Unit, Azienda Ospedaliera di Padova, Padova, Italy; ^11^Radiology Unit, ASST-Spedali Civili di Brescia, Brescia, Italy; ^12^Epidemiology Unit, Fondazione IRCCS Ca' Granda Ospedale Maggiore Policlinico, Milan, Italy; ^13^Department of Biomedical Sciences and Human Oncology, Section of Internal Medicine and Clinical Oncology, University of Bari Medical School, Bari, Italy; ^14^Department of Molecular Medicine, “Sapienza” University of Rome, Rome, Italy

**Keywords:** GLILD, CVID-ILD, CD21lo B cells, splenomegaly, autoimmune cytopenia, DLCO

## Abstract

**Background:** Granulomatous and Lymphocytic Interstitial Lung Diseases (GLILD) is a severe non-infectious complication of Common Variable Immunodeficiency (CVID), often associated with extrapulmonary involvement. Due to a poorly understood pathogenesis, GLILD diagnosis and management criteria still lack consensus. Accordingly, it is a relevant cause of long-term loss of respiratory function and is closely associated with a markedly reduced survival. The aim of this study was to describe clinical, immunological, laboratory and functional features of GLILD, whose combination in a predictive model might allow a timely diagnosis.

**Methods:** In a multicenter retrospective cross-sectional study we enrolled 73 CVID patients with radiologic features of interstitial lung disease (ILD) associated to CVID (CVID-ILD) and 125 CVID patients without ILD (controls). Of the 73 CVID-ILD patients, 47 received a definite GLILD diagnosis while 26 received a clinical-radiologic diagnosis of CVID related ILD defined as uILD.

**Results:** In GLILD group we found a higher prevalence of splenomegaly (84.8 vs. 39.2%), autoimmune cytopenia (59.6 vs. 6.4%) and bronchiectasis (72.3 vs. 28%), and lower IgA and IgG serum levels at CVID diagnosis. GLILD patients presented lower percentage of switched-memory B cells and marginal zone B cells, and a marked increase in the percentage of circulating CD21lo B cells (14.2 vs. 2.9%). GLILD patients also showed lower total lung capacity (TLC 87.5 vs. 5.0%) and gas transfer (DLCO 61.5 vs. 5.0%) percent of predicted. By univariate logistic regression analysis, we found IgG and IgA levels at CVID diagnosis, presence of splenomegaly and autoimmune cytopenia, CD21lo B cells percentage, TLC and DCLO percent of predicted to be associated to GLILD. The joint analysis of four variables (CD21lo B cells percentage, autoimmune cytopenia, splenomegaly and DLCO percent of predicted), together in a multiple logistic regression model, yielded an area under the ROC curve (AUC) of 0.98 (95% CI: 0.95-1.0). The AUC was only slightly modified when pooling together GLILD and uILD patients (0.92, 95% CI: 0.87-0.97).

**Conclusions:** we propose the combination of two clinical parameters (splenomegaly and autoimmune cytopenia), one lung function index (DLCO%) and one immunologic variable (CD21lo%) as a promising tool for early identification of CVID patients with interstitial lung disease, limiting the use of aggressive diagnostic procedures.

## Introduction

Common Variable Immunodeficiency (CVIDs) is the most commonly diagnosed (1), clinically relevant primary antibody deficiency characterized by both infectious and non-infectious complications. The introduction of intravenous or subcutaneous, immunoglobulin replacement therapy has markedly decreased morbidity and mortality due to infection (2, 3). In contrast, non-infectious complications, such as autoimmune manifestations, cytopenias, inflammation, lung disease, lymphoproliferation, and malignancies result increased, involving almost 70% of patients (4). The presence of non-infectious complications is associated with more severe prognosis and reduced quality of life (5–7).

Up to 90% of CVID patients may develop lung complications such as infection-related, immune-mediated and neoplastic diseases (8). Among these, Granulomatous and Lymphocytic Interstitial Lung Diseases (GLILD) is a severe non-infectious complication, reported in around 8–20% of cases (9, 10). GLILD has been defined as “a distinct clinico-radio-pathological ILD occurring in patients with CVID, associated with a lymphocytic infiltrate and/or granuloma in the lung, and in whom other conditions have been considered and where possible excluded” (9). It is a relevant cause of long-term lung damage and impairment of respiratory function and it is closely associated with poor clinical outcomes (5, 8, 11, 12) At present, although the pathogenesis of GLILD is still far from being understood, it may be considered as a manifestation of immune dysregulation (13), as also underlined by the increased frequency of other immune-mediated CVID complications in GLILD patients (14).

Based on UK-PID Network Consensus, current diagnostic recommendations in the suspicion of GLILD include chest CT scan, lung function tests (PFTs), bronchoscopy and a surgical lung biopsy, this latter mandatory to put a definite diagnosis (9). Several epidemiologic studies have underlined that the risk of performing a lung biopsy is clinically relevant and this risk increases with age, disease severity, or comorbidities (15). Moreover, GLILD in some cases may be misdiagnosed as granulomatous lung disease of other nature.

The possibility to define clinical, laboratory and radiological parameters that may identify CVID patients at high risk for GLILD development or allow for early diagnosis, might limit the use of lung biopsy and related risks and will potentially ameliorate affected patients' prognosis.

In addition, the introduction of MRI may represent a reliable radiation-free technique for diagnosis and follow-up of GLILD patients (16, 17), associated with evaluation of the broncho-alveolar lavage (BAL) in terms of GLILD related markers such as inflammatory cytokines and lymphocyte subsets (18).

Over the last years, different data have been reported on GLILD patients, suggesting that they are characterized by reduced overall survival and tend to develop an immune dysregulation including splenomegaly, lymphoproliferation and autoimmune cytopenias (12, 19, 20). Kellner et al. reported that patients with chronic lung disease had lower T cell counts and increased prevalence of non-bacterial infections in addition to autoimmune cytopenia (7) whereas Mannina et al. defined hypersplenism and polyarthritis as strong risk factors for GLILD (21). Finally, Hartono et al. proposed a GLILD predictive model based on splenomegaly, CD21lo B cells percentage, autoimmune cytopenia and serum IgA levels (14).

Nonetheless, to date, there is a lack of well-defined clinical, laboratory, and radiological parameters that may identify a clinical phenotype of patients affected by GLILD or prone to its development. With the aim to overcome this gap, we undertook this multicenter observational retrospective study in order to describe clinical, immunological, laboratory, and radiological features of GLILD patients that may lead to the identification of specific features and possible biological predictors capable of allowing an early diagnosis in patients at high risk to develop ILD (14).

## Materials and Methods

### Study Population

We conducted a multicenter retrospective cross-sectional study in which we enrolled patients with a diagnosis of CVID with interstitial lung disease (defined as CVID-ILD) and without it (defined as controls) from 7 Italian adult Italian Primary Immune Deficiency Network (IPINET) referral centers (Rome, Treviso, Milan, Brescia, Naples, Cagliari, Bari). Each center provided at least one age-matched control for each CVID-ILD patient.

All participants were enrolled in the IPINET Registry. This study was approved by the local institutional review board and was performed in accordance with the Declaration of Helsinki. All participants signed the written informed consent form prior to inclusion in the study.

Inclusion criteria were:

1) CVID diagnosis according to the ESID registry working party (22) with at least 18 months of follow-up since diagnosis;

2) For the subgroup of CVID-ILD patients a chest HRCT scan consistent with ILD according to existing literature, a bronchoalveolar lavage excluding and infectious interstitial pneumonia and:

- Either CVID-ILD diagnosis based on video-assisted thoracoscopic surgery (VATS) or transbronchial biopsy, or on lymph nodal or other organ's biopsy excluding B-cell malignancy. This group was defined as **GLILD** (9, 19, 23)
- **Or** CVID-ILD diagnosis obtained by clinical, functional and radiologic evaluation, in which no suspicion of B cell malignancy could be raised, a lung biopsy for histological diagnosis was too dangerous, or refused by the patients, or resulted no conclusive for GLILD. Patients belonging to this group were defined as undefined Interstitial Lung Disease **(uILD)**.

All CVID-ILD patients received a final diagnosis of **GLILD** or **uILD** after a multidisciplinary team discussion involving experienced Clinical Immunologists, lung Radiologists, Pathologists, Pulmonologists, with participation of Hematologists and Infectious disease Specialists when required (24).

For CVID-ILD and controls, the following reports had to be available at enrollment: at least one HRCT scan, 2 abdominal ultrasounds, IgG, IgA, and IgM levels at diagnosis and at last follow up, clinical history regarding cancer, enteropathy, autoimmune cytopenia, lymphoproliferation, smoking status, CD19+, and B lymphocytes subsets.

### Data Collection

The retrospective examination of clinical records of all enrolled subjects (GLILD patients, uILD patients and controls) aimed to investigate:

- Demographic parameters (Age, sex, BMI, smoking status, age at CVID diagnosis, diagnostic delay);
-Clinical phenotypes according to the revised Chapel et al. classification (5);
-Presence or absence of splenomegaly (defined as a spleen enlargement confirmed by two abdominal ultrasound and/or CT scan and/or MRI repeated at least 12 months apart from each other according to the Radiologist performing the test), bronchiectasis, autoimmunity, cancer;
-Laboratory parameters: IgG, IgA, IgM at CVID diagnosis and at last follow-up visit; for IgG, trough level (IgGTL) has been considered under replacement therapy
-Lymphocyte subsets according to Euroclass classification (25)
-Route and dosage of immunoglobulin replacement therapy (IgRT)
-Lung function, including 1st second Forced Expiratory Volume (FEV1), Forced Vital Capacity (FVC), Total Lung Capacity (TLC), and gas transfer (DLCO). Data were expressed as percent of predicted, according to ATS guidelines.
-Lung HRCT scan picture

In addition, for GLILD patients:

- Histology, site of biopsy
- 6-min walking test (distance, symptoms/desaturation), when available
- Broncho-alveolar lavage fluid (BALF) flow cytometry results, when available.

### HRCT Analysis

Blind HRCT scan evaluation was performed by three lung radiologists in a subgroup of GLILD patients and controls, in order to compare airways and parenchymal abnormalities. The following parameters were registered, scored in terms of absence/presence: bronchiectasis, bronchial wall thickening, mucus plugging, and centrilobular nodules, solid nodular opacities, excavated opacities, ground glass opacities <5 mm and >5 mm, consolidations, Halo sign, linear opacities, signs of fibrosis, mosaic attenuation, emphysema, lymph nodes increase in number and/or size, lymph nodes calcifications. Moreover, with the limits due to the possible non-complete inclusion of the whole spleen and liver (in particular) parenchyma in the scan, evidence of splenomegaly at caudal sections of HRCT scan was registered. Differences were resolved by consensus. For GLILD patients, HRCT scan images used for comparison were all acquired at GLILD diagnosis or at least before GLILD treatment. The list of radiological findings was defined on the basis of existing literature and clinical experience (26, 27). The syllabus of the Fleischner Society was used as a cornerstone for the radiological terminology, since the correspondence between images and definitions is well defined and widely accepted (28).

### Statistical Analysis

We used Wilcoxon rank-sum (Mann-Whitney) or Kruskal-Wallis test to compare quantitative variables across two or more groups, respectively. We reported median and interquartile range (IQR) as descriptive statistics. Chi-squared and Fisher's exact tests were used for categorical variables. Univariate and multivariable logistic regression models were fitted to calculate odds ratios (OR), 95% confidence intervals (CI) and area under the curve (AUC) of receiver operating characteristic (ROC) curves. Variables entered in the multivariable model were chosen based either on clinical grounds and existing literature or on results of univariate models. Statistical analyses were performed with Stata 16 (StataCorp.2019).

## Results

We enrolled 73 CVID patients with radiologic features of CVID-ILD and 125 CVID patients without ILD (controls). Of the 73 ILD patients 47 received a definite GLILD diagnosis while 26 were classified as uILD.

All patients were regularly treated with adequate substitutive treatment using polyvalent IgGs. 77.6% of controls, 87.23% of GLILD and 88.46% of uILD were under subcutaneous replacement therapy (SCIg). A total of 104 out of 198 patients performed a genetic screening: 2 patients with ILD presented a TACI mutation, as well as 3 controls, and 5 patients with CVID-ILD presented a CTLA4 mutation (3 with histologic diagnosis of GLILD, 1 with a clinical-radiologic diagnosis of uILD). Other genetic variants were detected in 1 control, 3 GLILD and 2 ILD patients. The screening for CVID associated genes is currently ongoing.

Demographic parameters are summarized in Table 1. No statistically significant differences were detected for controls and CVID-ILD patients in terms of sex, age, age at CVID diagnosis, age at CVID onset, and diagnostic delay. When focusing on CVID-ILD patients, uILD patients showed older age at CVID onset and a more recent CVID diagnosis when compared to GLILD patients. Median age at enrollment was 46, 47, and 49.5 years for controls, GLILD and uILD, respectively. There was a prevalence of female sex between controls and CVID-ILD patients (56 vs. 70%, respectively); the percentage of female patients was lower in uILD than in GLILD, but without statistical significance. Moreover, there was no difference between groups in terms of body weight, BMI and smoking status. A further description of the CVID-ILD population is available in the Supplementary Material. We will first present the results of the comparison between the control group and the GLILD group; finally, we will discuss similarities and differences between the GLILD and uILD subgroups, focusing on the role of clinical predictors in the diagnostic process.

**Table 1:** Characteristics of the population.

	**Controls *n* = 125**	**GLILD *n* = 47**	**uILD *n* = 26**	***p value***	***p value***	***p value***
	**median (IQR)**	**median (IQR)**	**median (IQR)**	**(GLILD vs. ctrls)**	**(uILD vs. GLILD)**	**(uILD vs. ctrls)**
Sex F (*n*; %)	70 (56.0%)	33 (70.2%)	13 (50.0%)	0.11	0.12	0.66
Age (years)	46 (34–59)	47 (37–60)	49.5 (43–61)	0.96	0.31	0.44
Age at CVID onset	28 (13.0–38.0)	21 (13.0–36.0)	38.5 (18.0–48.0)	0.36	**0.02**	0.07
Age at CVID diagnosis	37 (26.0–46.0)	35 (27.0–46.0)	42 (33.0–52.0)	0.92	0.16	0.25
Diagnostic delay (years)	6 (2.0–13.0)	6 (2.0–16.0)	5.5 (3.0–10–0)	0.52	0.45	0.31
Years since CVID onset	18 (10.0–27.0)	18 (11.0–33.0)	14 (8.0–20.0)	0.40	**0.02**	0.12
Body weight (Kg)	67.0 (56.8–82.0)	62.0 (59.0–75.0)	61.5 (56.0–77.0)	0.26	0.76	0.11
BMI	24.6 (21.3–27.8)	23.7 (21.4–26.0)	22.7 (20.5–27.2)	0.26	0.97	0.14
Current or former smoker (*n*; %)	29 (24.4%)	14 (29.8%)	4 (15.4%)	0.55	0.25	0.44
Antibiotic prophylaxis (*n*; %)	32 (25.6%)	22 (46.8%)	7 (26.9%)	**0.0099**	0.09	0.63

### Clinical Phenotype

We then compared GLILD and controls in terms of clinical phenotypes according to Chapel et al. (5) (Table 2). Control group included a significantly higher percentage of patients presenting the “infection only” phenotype (70.4 vs. 2.12%, *p* < 0.0001), while the GLILD group was characterized by an increased frequency of the lymphoproliferation and cytopenia phenotypes (*p* < 0.0001). No difference was detected in terms of enteropathy and cancer, being cancer borderline higher in the GLILD group. When considering the different types of cancer, the only significant difference was registered in the prevalence of T and B clonal lymphoproliferative diseases (B cell Non-Hodgkin lymphomas and T-large granular lymphocyte leukemia T-LGLL). Interestingly, a clear difference was detected between GLILD and controls when comparing the prevalence of bronchiectasis (*p* < 0.0001), splenomegaly (*p* < 0.0001) and idiopathic thrombocytopenic purpura (*p* < 0.0001). Of note, 8 of 47 GLILD patients and 3 of 26 uILD had previously undergone splenectomy, due to autoimmune cytopenia. Moreover, 5 Evans' syndromes were identified in GLILD, 1 in controls, none in uILD. In line with the higher prevalence of bronchiectasis, GLILD patients more frequently underwent antibiotic prophylaxis (*p* < 0.0001), that was almost performed with azithromycin 250 mg/die for 3 consecutive days per week, while in only 2 patients (belonging to the control group) with trimethoprim/sulfametoxazole (Table 1).

**Table 2:** Chapel's phenotypes I-IV and other disease-related complications.

	**Controls *n* = 125**	**GLILD *n* = 47**	**uILD *n* = 26**	***p value***	***p value***	***p value***
	***n* (%)**	***n* (%)**	***n* (%)**	**(GLILD vs. ctrls)**	**(uILD vs. GLILD)**	**(uILD vs. ctrls)**
Infections only (I)	88 (70.4)	1 (2.1)	8 (30.8)	**<0.0001**	**<0.001**	**<0.001**
Cytopenia (II)	13 (10.4)	29 (61.7)	8 (30.8)	**<0.0001**	**0.015**	**0.012**
Lymphoproliferation (III)	28 (22.4)	43 (91.5)	16 (61.5)	**<0.0001**	**0.004**	**<0.0001**
Enteropathy (IV)	14 (11.2)	9 (19.1)	6 (23.1)	0.20	0.76	0.11
Cancer	18 (14.4)	12 (25.5)	4 (15.4)	0.086	0.38	1.0
B-cell lymphoma	5 (4.0)	5 (10.6)	2 (7.7)	0.097	0.68	0.41
B-cell Lymphoma & T-LGLL	5 (4.0)	9 (19.1)	3 (11.5)	**0.001**	0.40	0.14
Splenomegaly	49 (39.2)	39 (84.8)	20 (70.0)	**<0.0001**	0.52	**<0.0001**
Bronchiectasis	35 (28.0)	34 (72.3)	15 (57.7)	**<0.0001**	0.20	**0.003**
ITP	8 (6.4)	26 (55.3)	8 (30.8)	**<0.0001**	**0.044**	**0.0018**
AI cytopenia (AIHA+ITP)	13 (10.4)	28 (59.6)	8 (30.8)	**<0.0001**	**0.027**	**0.012**
Autoimmunity	35 (28.0)	32 (68.1)	11 (42.3)	**<0.0001**	**0.047**	0.16

*AI cytopenia, history of ITP and/or AIHA*.

### Ig Serum Levels, IgG Trough Level, and Ig Replacement Therapy

IgG serum levels at the time of CVID diagnosis were found significantly lower both in GLILD (IgG 241.0 mg/dl, IQR 79.0-382.0) and in uILD (230.0 mg/dl, IQR 109-307) than in controls (349 mg/dl, IQR 167.0-451.0) (*p* < 0.05). The same was observed for IgA (**GLILD** 8.0 mg/dl, IQR 1.2-21.0; **uILD** 6.0 mg/dl, IQR 5.0-9.5; **controls** 17.0 mg dl, IQR 6.0-29.5; *p* < 0.01). No difference was found in IgM levels at diagnosis and at last follow-up. (**GLILD** 19.0 mg/dl, IQR 4.0-35.0; **uILD** 9.5 mg/dl, IQR 5.0-30.0; **controls** 21.5 mg/dl, IQR 10.0-41-0 at diagnosis) (*p* > 0.05); (**GLILD** 20.0 mg/dl, IQR 4.0-50.0; **uILD** 15.5 mg/dl, IQR 4.5-41; controls 22.0 mg/dl, IQR 5.0-46.0 at last follow-up (*p* > 0.05). Only 2 patients presented an increase in polyclonal IgM levels after CVID-ILD diagnosis, one with GLILD and one with uILD. The difference in IgA serum level was confirmed at last FU; IgG trough levels were similar in GLILD, uILD and controls (**GLILD** 799.2 mg/dl, IQR 677.5-933.5; **uILD** 833.0 mg/dl, IQR 733.0-944.5; **controls** 796.5 mg/dl, IQR 669.0-937.5) (*p* > 0.05) (Supplementary Figure 1).

GLILD and uILD patients required higher dosage of IgRT than controls to achieve similar IgG trough levels (**GLILD** 400.0 mg/kg -IQR 350-480; **uILD** 402.0 mg/kg, IQR 380-500; **controls** 365.4 mg/kg -IQR 274.3-444.0) (*p* < 0.05) (Supplementary Figure 1). No differences were found between GLILD and uILD for any of the Ig-related measures. There was no difference in route of Ig administration between groups.

### Lymphocytes Subsets

CVID patients with and without GLILD were then compared analyzing B and T cell subsets before immunosuppressive treatment. There were no differences in lymphocytes absolute count and percentage. CD19+ B cell absolute value and percentage was similar in the two groups; the prevalence of patients with <1% of circulating B cells was also superimposable (16.67% GLILD, 12.0% controls). 60.6% of GLILD patients and 44.09% of controls presented <2% of switched-memory B cells (SmB), with no significant difference; however, when comparing SmB percentage of B cells, GLILD patients presented lower values than controls (*p* < 0.05). GLILD patients also showed a lower percentage of marginal zone B cells (MZB) than controls (*p* < 0.05). No differences were found in distribution of plasmablasts, naïve and transitional B cells. Of note, GLILD patients showed a significant increase in the percentage of circulating CD21lo B cells compared to controls (*p* < 0.0001) (Table 3).

**Table 3:** B Lymphocytes subsets.

	**Controls *n* = 125**	**GLILD *n* = 47**	**uILD *n* = 26**	***p value***	***p value***	***p value***
	**Median (IQR)**	**Median (IQR)**	**Median (IQR)**	**(GLILD vs. ctrls)**	**(uILD vs. GLILD)**	**(uILD vs. ctrls)**
Lymphocytes %	29.0 (22.3–37.6)	27.8 (21.8–39.4)	29.0 (26.0–35.0)	0.87	0.54	0.93
Lymphocytes count	2.05 (1.4–2.6)	1.54 (0.99–2.57)	1.87 (1.10–2.30)	**0.02**	0.67	0.44
CD19+ B cells (% of lymphocytes)	7.0 (3.0–12.6)	6.0 (3.0–10.0)	4.0 (2.0–11–0)	0.91	0.17	0.32
Naïve (% of B cells)	72 (56.6–86.0)	81.2 (54.2–88.2)	75.9 (52.0–81.2)	0.48	0.10	0.63
Switched memory (% of B cells)	2.5 (1.0–6.6)	1.3 (0.1–5.0)	3.0 (0.6–5.9)	**0.043**	0.20	0.71
Marginal zone (% of B cells)	11.1 (2.4–24.6)	3.5 (1.3–11.0)	8.0 (5.0–16.7)	**0.043**	0.27	0.48
Transitional (% of B cells)	1.0 (0.2–2.5)	0.6 (0.0–4.0)	2.7 (0.6–7.5)	0.94	0.14	0.09
Plasmablasts (% of B cells)	0.1 (0.0–0.8)	0.3 (0.0–1.1)	0.1 (0.0–1.2)	0.11	0.47	0.66
CD21lo (% of B cells)	3.9 (1.9–7.7)	14.2 (10.1–30.0)	6.0 (2.8–29.3)	**<0.0001**	0.24	0.051

*B cells sub-populations are identified according to EUROclass study: Naïve IgD^+^IgM^+^CD27^−^; Switched memory IgD^−^IgM^−^CD27^+^; Marginal zone IgD^+^IgM^+^CD27^+^; Transitional CD38^++^IgM^high^; Activated CD21^low^CD38^low^; Plasmablasts CD38^+++^IgM^−^(25)*.

When analyzing T cells, GLILD patients presented a lower percentage of CD8+ T cells if compared to controls (*p* < 0.01), with an increased CD4/CD8 ratio (*p* < 0.05). Of note, GLILD patients presented a borderline significant expansion of CD3CD8CD57+ large T granular lymphocytes (*p* = 0.06), becoming significant when pooling together uILD and GLILD subgroups (*p* < 0.01) (Supplementary Table 1).

### Lung Function

As lung function parameters, according to data availability in clinical records, we considered 1st second Forced Expiratory Volume (FEV1), Forced Vital Capacity (FVC), Total Lung Capacity (TLC), and gas transfer (DLCO). Data were collected before starting any GLILD-specific treatment, as absolute values and percent of predicted. GLILD patients showed a significantly lower FEV1, FVC, TLC, and DLCO compared to controls. When adjusted for disease duration, differences in FEV1 (*p* = 0.006), FVC (*p* < 0.001), TLC% (*p* 0.001), and DLCO% (*p* < 0.001) were still significant between GLILD and controls (Table 4).

**Table 4:** Lung function parameters.

	**Controls *n* = 125**	**GLILD *n* = 47**	**uILD *n* = 26**	***p value***	***p value***	***p value***
	**Median (IQR)**	**Median (IQR)**	**Median (IQR)**	**(GLILD vs. ctrls)**	**(uILD vs. GLILD)**	**(uILD vs. ctrls)**
FEV1 (% of predicted)	102 (89–111)	88 (72–105)	103 (89–110)	**0.02**	**0.03**	0.94
				**0.006**	0.15	0.35
FVC (% of predicted)	104 (92–116)	88 (72–103)	104 (93–113)	**<0.001**	**0.01**	0.72
				**<0.001**	**0.01**	0.40
TLC (% of predicted)	102 (94–108)	87 (75–102)	93 (87–104)	**<0.001**	0.32	**0.03**
				**0.001**	0.29	**0.05**
DLCO (% of predicted)	83 (75–97)	61 (52–80)	73 (65–86)	**<0.001**	**0.008**	**0.02**
				**<0.001**	**0.02**	0.07

*For each cell: Upper p-value from Wilcoxon-Mann-Whitney test. Lower p value from linear regression models adjusted for disease duration (difference between age at enrolment and age at CVID onset)*.

### HRCT

Since the presence of specific CVID-ILD features represented an Inclusion Criteria both for GLILD and uILD group, there were no differences between these two groups at HRCT scan. HRCT scan evaluation by three experienced lung radiologists was then performed in a subgroup of 26/47 GLILD patients and 26/125 controls, in order to confirm the appropriate selection. Airways and parenchymal abnormalities were evaluated (Supplementary Table 2). As expected, a statistically significant difference in favor of GLILD patients was detected in terms of Bronchiectasis (*p* < 0.05), solid nodular opacities (*p* < 0.01), ground glass opacities <5 mm (*p* < 0.01) and >5 mm (*p* < 0.001), consolidations (*p* < 0.0001), halo sign (*p* < 0.0001), linear opacities (*p* < 0.0001), signs of fibrosis (*p* < 0.0001), mosaic attenuation (*p* < 0.05), lymph nodes increase in number (*p* < 0.001) and size (>1 cm) (*p* < 0.0001, absent in control group). Lymph nodes calcifications and excavated opacities were present in only one and two GLILD patients, respectively, and absent in controls. Moreover, detection of splenomegaly at caudal sections of HRCT scan was significantly higher in GLILD patients (*p* < 0.05), being 2/26 GLILD patients already splenectomized at the time of imaging acquisition; no difference was found in the prevalence of hepatomegaly in the same sections between the two groups. No significant difference was recorded when comparing prevalence of bronchial wall thickening, mucus plugging and centrilobular nodules and signs of emphysema.

### Broncho-Alveolar Lavage

All patients underwent bronchoscopy for microbiologic analysis of BALF during diagnostic work-up. BALF cell differential count was available for 21 patients (all with defined GLILD). Mean lymphocytes percentage was 31.42% (SD 24.9), with a median value of 26% (IQ range 18.5–38%) and 15/21 presented a lymphocytosis higher than 20%. When lymphocytes subpopulations analysis was available, mean CD4/CD8 ratio (19 patients) was 2.23 (SD 1.93), median was 1.58 (IQ range 0.53–3.6); 5 patients presented a CD4/CD8 ratio >3.5, as per sarcoidosis diagnostic criteria (18) and 7 > 3.0; in 7 patients ratio was reduced (<1.4). B cell percentage was available for 15 patients, showing a mean 6.82% (SD 5.35), with a median of 6.0% (IQ range 2-10). Five of these patients underwent B cell subpopulations analysis, all showing more than 75% CD21lo B cells.

### Logistic Regression Models and ROC Curves

As shown in Table 5, DLCO percent of predicted and CD21lo B cells percentage, history of autoimmune cytopenia, and presence of splenomegaly, presented a high power in predicting GLILD.

**Table 5:** Univariate logistic regression analysis and area under ROC curve for different possible GLILD predictors.

	**GLILD vs. Controls**	**Odds Ratio**	***p value***	**AUC**
	***n***	**(95% C.I.)**		
IgA at diagnosis (mg/dl)	47 vs. 125	0.97 (0.95–0.99)	0.008	0.65
IgG at diagnosis (mg/dl)	47 vs. 125	0.997 (0.995–0.999)	0.048	0.60
CD21lo B cells %	29 vs. 100	1.099 (1.05–1.15)	<0.001	0.78
FVC (% of predicted)	44 vs. 99	0.96 (0.94–0.98)	<0.001	0.71
DLCO (% of predicted)	38 vs. 75	0.94 (0.91–0.96)	<0.001	0.80
TLC (% of predicted)	34 vs. 64	0.95 (0.92–0.98)	0.001	0.71
AI Cytopenia (ITP, AIHA)	47 vs. 125	12.69 (5.60–28.77)	<0.001	0.75
ITP	47 vs. 125	18.11 (7.23–45.37)	<0.001	0.74
Splenomegaly	47 vs. 125	8.86 (3.68–21.36)	<0.001	0.73

*n, number of observations. ITP, AIHA: history of ITP and/or AIHA*.

**Figure 1:**
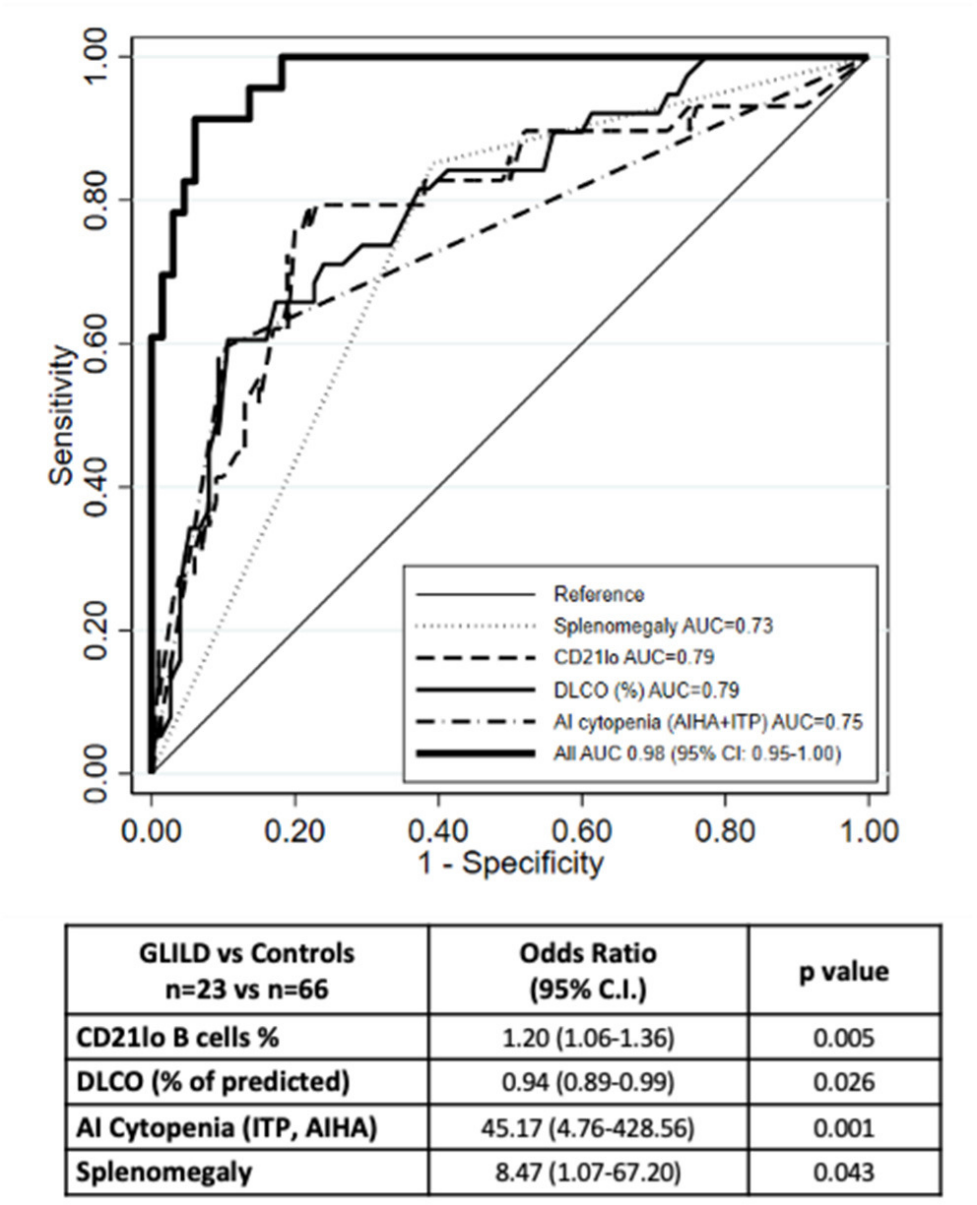
ROC curve of the multiple logistic regression model. The ROC curve of the multiple logistic regression model underlies an AUC of 0.98. Number of observations 89 (23 GLILD and 66 controls). The graph also shows the ROC curves for the logistic regression analysis of the single variables.

The final multivariate model including the above-mentioned parameters allowed us to reach a better predictive performance. The joint analysis of these four variables together in a multiple logistic regression model yielded an AUC of 0.98 (95% CI: 0.95-1.0) (Figure 1). The corresponding equation is:

$$\begin{array}{l} \text{Odds }(\text{GLILD})=\text{exp}[-0.530+(2.136\times \text{Sp})+(0.1838\times \text{CD})\\ \;-(0.063\times \text{DL})+(3.810\times \text{ AI})] \end{array}$$

where Sp = splenomegaly (yes = 1), CD = CD21lo (%), DL =DLCO (%) and AI = autoimmune cytopenia (yes = 1). Hence the predicted probability of GD1 can be calculated as: 100 x [odds(GLILD)/[1 + odds(GLILD)].]

When we compared model predictions with actual diagnoses, we observed that, when the probability predicted by the equation was <50%, there were only four subjects with GLILD out of 67 (6.0%); when the predicted probability was 50% or more, the observed frequency was 86.4% (19/22). This means that in order to have a strong indication of the presence of GLILD in a given subject, the probability predicted from the algorithm should be quite high (50% or more).

### GLILD and Other uILD Patients

As recapitulated in the previous tables and figures, uILD and GLILD patients did not differ only for the histologic evidence of granuloma. However, uILD patients presented many similarities and few differences when compared to the GLILD group. In terms of demographics, uILD patients appeared to have later CVID onset and a shorter history of disease (Table 1). In terms of clinical phenotypes, uILD patients presented a lower prevalence of cytopenia and lymphoproliferation compared to GLILD, but the prevalence was still significantly higher than in controls; the prevalence of bronchiectasis and splenomegaly was similar to GLILD (Table 2). When moving to immunologic parameters, uILD patients showed a significant reduction in IgG and IgA levels at CVID diagnosis if compared to controls, similarly to the GLILD group, and as for GLILD required higher dosage of IgRT than controls in order to achieve similar IgG trough levels (Supplementary Figure 1). The lower lymphocyte count and higher percentage of CD21lo % of B cells compared to controls were confirmed in uILD as shown for GLILD patients, despite being less significant. uILD patients also showed a significantly lower percentage of circulating CD4+ T cells (Table 3 and Supplementary Table 2).

GLILD patients presented a worse respiratory function if compared to uILD patients, with lower values of all considered parameters and a significant difference, in particular, when considering FVC and DLCO percent of predicted (Table 4). However, both DLCO and TLC of uILD patients resulted to be significantly lower than controls.

In conclusion this uILD group, despite presenting a shorter history of disease and a lower prevalence of autoimmune cytopenias, appeared to be quite similar to the GLILD group when considering the main putative predictors of CVID-ILD. This is confirmed by the ROC curve of the multivariate analysis including all CVID-ILD patients, showing an AUC of 0.92 (Supplementary Figure 2) when considering the same clinical and immunologic parameters in the GLILD population only, and by the history of GLILD specific treatment (Supplementary Table 3) showing that the prevalence of immune-suppressive treatment was higher in GLILD (*p* < 0.01) but, when specific indication was determined by interstitial lung disease, it was no more significantly different between GLILD and uILD patients (51.0 vs. 26.9%; *p* = 0.052).

## Discussion

CVID-ILD represents a relevant clinical issue in the management of CVID patients. Solid data regarding pathogenesis, diagnostic and prognostic markers, as well as treatment strategies are currently lacking. Moreover, different definitions such as CVID-ILD and GLILD are used in literature, whose borders and subsequent clinical implications are not clearly defined. For example, recent studies regarding clinical predictors of CVID-ILD did not routinely distinguish patients according to the presence or absence of a histologic confirmation of GLILD, despite using GLILD as nomenclature, while published retrospective cohorts exploring therapeutic approaches tend to focus on histologically defined ILDs (7, 12–14, 20, 21, 23). At present, retrospective studies on single-center or multicenter cohorts still constitute the main sources of information for Clinicians. To our knowledge, this is the first Italian multicenter study on CVID patients affected by interstitial lung disease (ILD). In our study we aimed to investigate clinical predictors and course of patients with a definite diagnosis of GLILD and those with similar/identical radiologic features not fulfilling the most accredited criteria for GLILD, that we named as undefined ILD (uILD) (9, 20, 29). We first compared the definite GLILD group with a control group of CVID patients without signs of interstitial lung disease.

Using the Chapel classification of CVID main clinical features, we found in the GLILD group an increased frequency of the lymphoproliferation and cytopenia phenotypes and a higher prevalence of clonal lymphoproliferative diseases when pooling together B cell lymphomas and T-LGLL. GLILD patients also showed a higher prevalence of splenomegaly and autoimmunity, mainly due to autoimmune cytopenias, in line with previously published data (14, 20, 21). Differently from what reported by Mannina et al. (21) polyarthritis was not registered at all in our CVID-ILD and controls, as in Versky's cohort. Interestingly, a higher prevalence of bronchiectasis was identified between our GLILD patients, which explains also the more frequent use of antibiotic prophylaxis, compared to previously published data. Low IgA serum levels in CVID have been reported as risk factors for development of bronchiectasis (30). Considering that GLILD patients have lower IgA levels when compared to controls, this could be a plausible explanation for the increased presence of bronchiectasis in our cohort. It does not seem related, instead, to CVID duration, since this was not different between cases and controls.

Immunological evaluation of our cohort of GLILD patients confirmed lower IgG and IgA levels at diagnosis, together with a requirement for a higher dose of IgRT in order to reach IgG trough levels similar to controls. GLILD patients presented lower percentage of switched-memory B cells and marginal zone B cells, as shown by Mannina et al. (21). Finally, they showed a significant increase in the percentage of circulating CD21lo B cells as reported by Hartono et al. (14).

As described in other cohorts, our GLILD patients also had lower lymphocyte counts, with a reduction in CD8+ T cells and an increase in CD4/CD8 ratio when compared to controls. Similar findings were recently reported by Kellner et al. (7) and were associated with increased frequency of pneumonia, herpes viruses and fungal infections. Our study on the other hand was not designed to compare infections rate and type between CVID-ILD and controls. However, we found a higher prevalence of bronchiectasis, smB cells reduction, lower IgG and IgA levels at CVID diagnosis, together with a more frequent use of antibiotic prophylaxis in the GLILD group. It is also to be considered that ILD patients, as in our cohort, might more frequently receive steroids and immune-suppressive drugs both for ILD and associated autoimmune complications (e.g., AI cytopenia) which may also increase the susceptibility to infections (20).

Of note, our CVID-ILD patients presented a significant expansion of CD3CD8CD57+ large T granular lymphocytes, in few patients recognized as T-LGLL; this might be related to splenomegaly/splenectomy, but the same population and T-LGLL itself are known to be related to autoimmune rather than cancer-related manifestations and deserves further investigation (31).

The study of lung function showed in our GLILD cohort lower FEV1%, FVC%, TLC%, and DLCO% compared to controls, with statistically significant differences particularly in FVC%, TLC%, and DLCO%. These data, except for TLC were already reported by Mannina et al. (21) but are quite far from what reported by Hartono et al. (14) We hypothesize that the difference in lung function between ours and other cohorts might rely on different length of CVID history, diagnostic delay, or other population-specific variables such as BMI, coexistence of asthma/COPD and related therapy.

By univariate logistic regression analysis, we explored the performance of the above discussed variables in predicting GLILD diagnosis, and we found presence of splenomegaly and autoimmune cytopenias, IgG and IgA levels at CVID diagnosis, CD21lo B cells percentage, TLC, FVC, and DLCO percent of predicted all presenting low *p* values. Most of these variables had already been somehow evaluated in previously proposed predictive models for GLILD. We finally defined a predictive model including autoimmune cytopenias, splenomegaly, DLCO percent-of-predicted, and CD21lo B cells percentage, that produced an area under the ROC curve of 0.98. Previously proposed models included either cytopenia, splenomegaly and CD21lo% without any lung function parameter (14) or hypersplenism and FVC% but without any immunologic marker (21). Conversely, our predictive model pools together two clinical variables, CD21lo B cells percentage as immunologic and DLCO% as lung function parameter.

We strongly agree with Mannina et al. (21) on the importance of including a lung-related parameter in a tool that is designed to help diagnosing a systemic disease with a focus on lung interstitium. DLCO and FVC are the key measures in the follow-up and treatment indication of ILDs. DLCO, compared to FEV1, is less affected by concomitant broncho-active treatment. The sensitivity of HRCT at detecting early signs of ILD is well recognized, as shown by Verbsky et al. (20) but still there is lack of evidence-based data on how and when to treat CVID-ILD patients. Hence, it is reasonable to take into account lung function decline when defining treatment indication, provided that ILD is the actual indication for treatment (20).

On the other hand, it is reasonable to include CD21lo B cells in a predictive model for GLILD, as this subset of B cells has been previously reported to be expanded in CVID patients, expressing pro-inflammatory chemokine receptors predicting the ability of tissue homing like the bronchoalveolar space have the capacity to home to sites of inflammation (32). We indeed reported data on BALF analysis showing that, in all 5 GLILD patients where B cell subpopulations analysis was available, more than 75% of these cells were actually CD21lo B cells. Moreover, in agreement with existing literature, we found a significant BALF lymphocytosis without univocal behavior of CD4/CD8 ratio, and with an increase of B cell percentage in a subgroup of patients.

Broncho-alveolar lavage is routinely used in GLILD work-up for microbiological differential diagnosis. However, BALF findings might also provide data on the different pathogenetic mechanisms and patients' prognosis (18, 33). Thus, we may hypothesize that a more widespread use of BALF analysis and uniformed lymphocyte phenotyping might help to dissect the ongoing lung inflammatory processes (e.g., presence of a CD4+ alveolitis, B cell increase and activation, mediators potentially acting as activity biomarkers) and to potentially define tailored treatments that, at present, are provided only by histologic evaluation.

Finally, as also reported in previous studies, our GLILD and uILD sub-cohorts showed definitely more similarities than differences, as confirmed by the multivariate logistic regression; when we applied our algorithm to the uILD cohort, we identified a subgroup of uILD patients with high probability of GLILD despite the lack of a histologic diagnosis. This raises the question whether the histologic investigation is always mandatory or should be limited to specific cases. Histology is currently the gold standard for GLILD diagnosis. However, we hypothesize that a clinical-radiologic evaluation, in an appropriate multidisciplinary context and with the support of our proposed prediction model (under validation) might be enough for GLILD diagnosis in a proportion of cases, particularly with the aid of genetics and BALF results as possible histologic surrogate. Further studies are needed to confirm our hypothesis. Our study has several limitations, shared with previous study published on this topic, mainly due to the retrospective study design and to the non-univocal definition of CVID-ILD, which is yet an unsolved issue. Despite this, the strengths of this study are the numerous cohorts of GLILD and controls enrolled, the multicentric design and the multidimensional comparison between groups of patients. In conclusion, our findings highlight the strong need for prospective multicenter studies in the complex field of ILD in CVID in order to ameliorate diagnostic tools and prognosis for affected patients.

## Data Availability Statement

The raw data supporting the conclusions of this article will be made available by the authors, without undue reservation.

## Ethics Statement

The studies involving human participants were reviewed and approved by Comitato Etico delle Province di Treviso e Belluno. The patients/participants provided their written informed consent to participate in this study.

## Author Contributions

FC, CM, RS, IQ, and CA conceptualized the study. FC, CM, VL, APl, and RS designed the protocol study. GG, VS, HB, SG, APu, GL, GC, CiM, MC, GT, CaM, SD, MR, AV, and GF recruited patients and collected data. DC and FC did the statistical analysis. NL, SV, and MB performed the radiological analysis. FC, CM, MC, VL, DF, RS, and GS prepared the first draft of the manuscript. All authors reviewed the manuscript before publication.

## Conflict of Interest

The authors declare that the research was conducted in the absence of any commercial or financial relationships that could be construed as a potential conflict of interest. The handling editor declared a past collaboration with the author IQ.
