# Supplementary material for: Granulomatous Lymphocytic Interstitial Lung Disease (GLILD) in Common Variable Immunodeficiency (CVID): A Multicenter Retrospective Study of Patients From Italian PID Referral Centers
Source: Front Immunol. 2021 Mar 10;12:627423. doi: 10.3389/fimmu.2021.627423 (PMC7987811; doi:10.3389/fimmu.2021.627423)
Supplement: Supplementary file 1 [file Data_Sheet_1.docx]

**SUPPLEMENTARY MATERIAL**


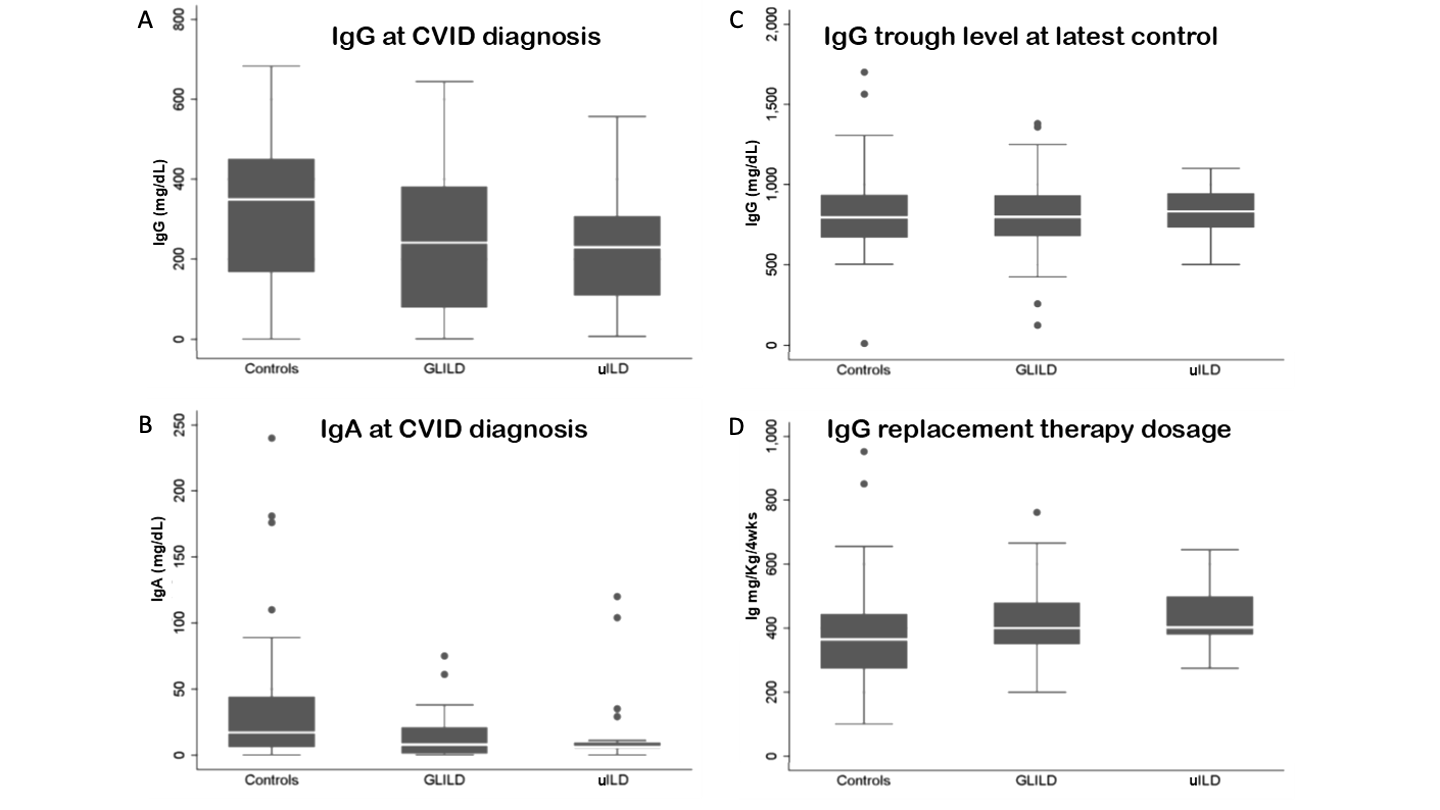


**SUPPLEMENTARY FIGURE S1: IgG and IgA at CVID diagnosis, IgG trough level and IgGRT dosage (mg/kg/4weeks) in Controls, GLILD and uILD patients.** (**Panel A and B**): IgG (p=0.04 and p=0.02) and IgA (p=0.003 and p=0.007) levels at CVID diagnosis were significantly lower in GLILD and in uILD patients (respectively) than in the control group. (**Panel C**): IgG trough level at the last available follow-up were not different between controls, GLILD and uILD patients. (**Panel D**): GLILD and uILD patients required higher dosage of IgRT than controls to achieve similar IgG trough levels (p=0.015 and p=0.014 respectively). No differences were found between GLILD and uILD for any of the Ig-related measures.

**SUPPLEMENTARY FIGURE S2**

**
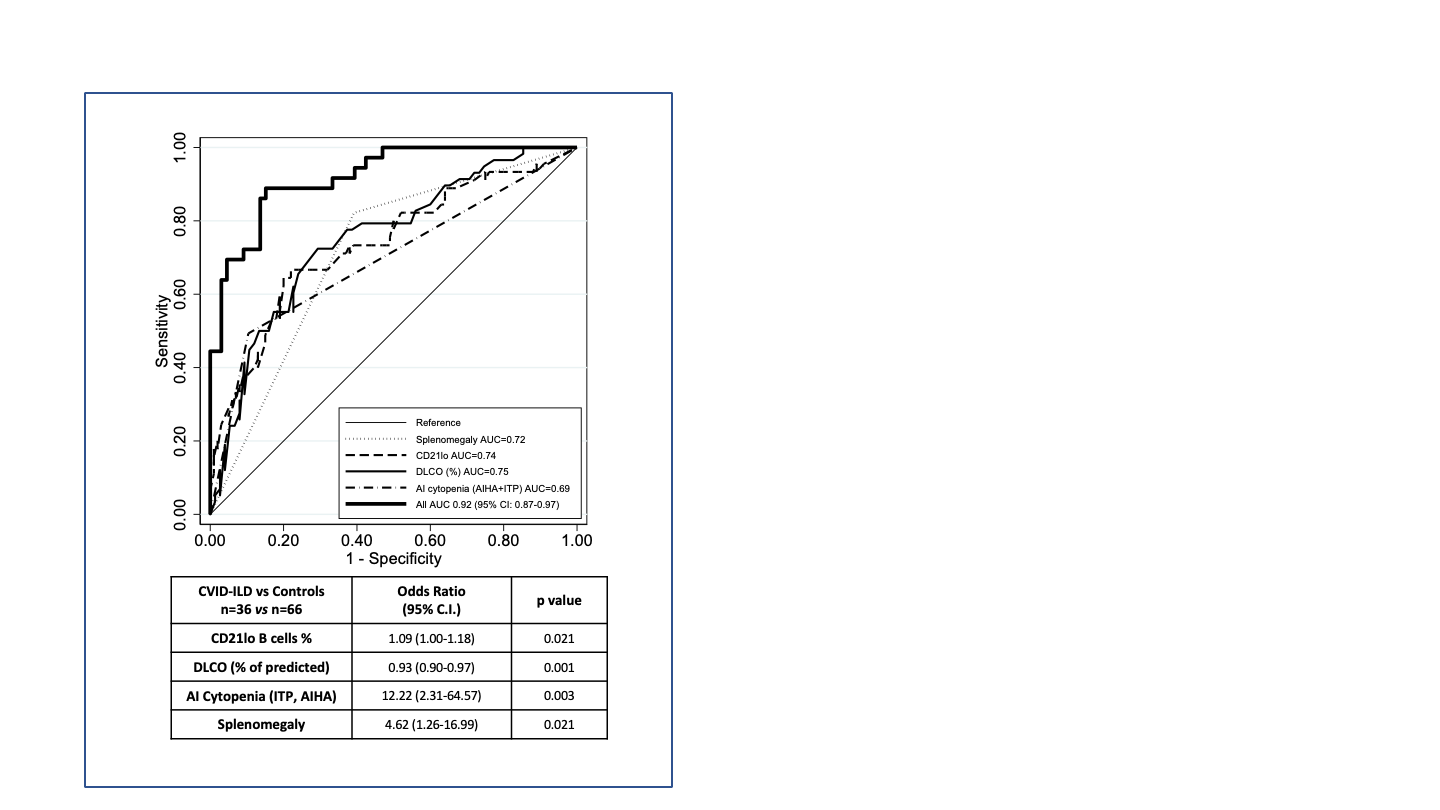
**

**SUPPLEMENTARY FIGURE 2 – ROC curve of the multiple logistic regression model including GLILD and uILD patients.** The ROC curve of the multiple logistic regression model underlies an AUC of 0.92. Number of observations 89 (23 GLILD + 13 uILD and 66 controls). The graph also shows the ROC curves for the logistic regression analysis of the single variables.

**Description of CVID-ILD population**

Of the 73 CVID-ILD patients, 47 received a GLILD diagnosis after histologic confirmation (lung 21 patients, lymph nodes 29 patients of which 4 below diaphragm, spleen 3, liver 5, bone marrow 9, other organs 6); of these, 22 patients had histologic evidence of granuloma and/or polyclonal lymphocytic infiltrate in biopsies of at least 2 different sites. 26 patients showed non necrotizing granulomas at histologic examination, while 39 presented features of lymphoid hyperplasia. 18 patients were active or former smokers, 5 had a clinical history of possible but not definite professional exposure. 8 had received a previous diagnosis of sarcoidosis before CVID diagnosis. Of 26 uILD patients, 5 underwent non-diagnostic biopsies, while 16 refused the procedure; for 5 patients an invasive approach was too dangerous according to the multidisciplinary team discussion.

CVID-ILD patients received steroids and/or immune-suppressive treatment in 68.5% of cases (p<0.01 GLID *vs* uILD). CVID-ILD was the treatment indication in 42.5% (p=0.0525 GLILD *vs* uILD) (Supplementary Table 3). In almost all other cases treatment indication was an autoimmune Cytopenia (either ITP or AIHA). The adopted therapeutic strategies are recapitulated in Supplementary Table 4. In addition, 5 patients received Rituximab plus chemotherapy due to hematological malignancies. One patient underwent bilateral lung transplantation due to end stage lung disease; definite histologic diagnosis of GLILD was obtained on explanted lungs. Finally, 2 GLILD patients died despite specific treatment, one (with a previous history of treated Burkitt lymphoma) due to infectious complications, the other due to the onset of a very aggressive Burkitt Non-Hodgkin Lymphoma.

**Application of the proposed algorithm to the uILD population**

When we applied the algorithm (developed among controls and GLILD cases) to controls and uILD cases, we observed that GLILD were 8/71 (11.3%) when the predicted probability was <50% and 5/8 (62.5%) when the algorithm probability was ≥50%. The meaning of the proposed algorithm is to avoid unnecessary invasive bioptic approaches; when applied to the uILD cohort, the algorithm would allow to have a GLILD diagnosis for 5 of the 13 uILD patients whose DLCO and CD21lo data were fully available. On the other hand, it would suggest to be more aggressive for the 8 uILD patients with a predicted probability <50%. Hopefully, the algorithm will be further validated in larger cohorts.

**Supplementary Tables**

**SUPPLEMENTARY TABLE 1. T cells subsets**

|  | **Controls n=125** | **GLILD n=47** | **uILD n=26** | ***p value*** | ***p value*** | ***p value*** |
| --- | --- | --- | --- | --- | --- | --- |
|  | **median (IQR)** | **median (IQR)** | **median (IQR)** | **(GLILD *vs* ctrls)** | **(uILD *vs* GLILD)** | **(uILD *vs* ctrls)** |
| **CD3+ %** | 77 (71.0-82.0) | 79 (72.0-84.0) | 79.5 (73.5-85.5) | 0.57 | 0.57 | 0.58 |
| **CD4+ %** | 41.0 (35.0-53.0) | 46.4 (36.0-50.9) | 38.8 (25.9-47.0*)* | 0.71 | **0.04** | 0.23 |
| **CD4+ (cells/µl)** | 540.5 (530.5-831.6) | 540.5 (48.0-717.9) | 537.8 (455.0-852.6) | 0.21 | 0.94 | 0.66 |
| **CD8+ %** | 34.0 (26.0-42.0) | 30.0 (26.0-35.0) | 34.0 (22.0-37.3) | **0.042** | 0.23 | 0.99 |
| **CD4/CD8 ratio** | 1.2 (0.8-1.7) | 1.6 (1.2-2.3) | 1.2 (0.6-2.2) | **=0.002** | 0.15 | 0.20 |
| **CD3+CD8+CD57+ %** | 10.0 (4.0-17.0) | 13.1 (7.0-25.0) | 13.9 (10.3-40.0) | 0.058 | 0.44 | 0.15 |
|  | **Controls n=125** | **GLILD+uILD n=73** | ***p value*** |  |  |  |
|  | **median (IQR)** | **median (IQR)** |  |  |  |  |
| **CD3+CD8+CD57+ %** | 10.0 (4.0-17.0) | 13.0 (7.3-28.0) | **=0.01** |  |  |  |

**SUPPLEMENTARY TABLE 2. HRCT findings GLILD *vs* controls**

|  | **Controls n=26** | **GLILD n=26** | ***p value*** |
| --- | --- | --- | --- |
| **HRCT findings** | **n (%)** | **n (%)** | **(GLILD *vs* ctrls)** |
| **Bronchiectasis** | 12 (46.1) | 21 (80.7) | **0.02** |
| **Bronchial wall thickening** | 9 (34.6) | 12 (46.1) | 0.57 |
| **Mucus plugging and centrilobular nodules** | 6 (23.0) | 7 (26.9) | 0.99 |
| **Solid nodular opacities** | 8 (30.7) | 19 (73.0) | **0.005** |
| **Excavated opacities** | 0 (0.0) | 2 (7.6) | 0.49 |
| **Ground glass opacities <5 mm** | 6 (23.0) | 18 (69.2) | **0.0019** |
| **Ground glass opacities >5 mm** | 6 (23.0) | 20 (76.9) | **0.0002** |
| **Consolidations** | 3 (11.5) | 19 (73.0) | **<0.0001** |
| **Halo sign** | 1 (3.8) | 17 (65.3) | **<0.0001** |
| **Linear opacities** | 5 (19.2) | 21 (80.7) | **<0.0001** |
| **Signs of fibrosis** | 1 (3.8) | 15 (57.7) | **<0.0001** |
| **Mosaic attenuation** | 4 (15.3) | 13 (50.0) | **0.0167** |
| **Signs of emphysema** | 3 (11.5) | 6 (23.0) | 0.46 |
| **Lymph nodes increase in number** | 3 (11.5) | 15 (57.7) | **0.001** |
| **Lymph nodes increase in size (>1 cm)** | 0 (0.0) | 13 (50.0) | **<0.0001** |
| **Lymph nodes calcification** | 0 (0.0) | 1 (3.8) | 0.99 |

**SUPPLEMENTARY TABLE 3. CVID-ILD treatment**

|  | **All CVID-ILD n=73** | **GLILD n=47** | **uILD n=26** |  |
| --- | --- | --- | --- | --- |
|  | **n (%)** | **n (%)** | **n (%)** | ***p value*** |
| **Treatment** | 50 (68.5) | 40 (85.1) | 10 (38.5) | 0.005 |
| **Treatment indication: CVID-ILD** | 31 (42.5) | 24 (51.0) | 76 (26.9) | =0.0525 |

**SUPPLEMENTARY TABLE 4. Drugs used for CVID-ILD treatment**

| **Treatment** | **All indications, n=73** | **CVID-ILD indication, n=73** |
| --- | --- | --- |
|  | **n (%)** | **n (%)** |
| **Steroids** | 40 (54.8) | 24 (32.9) |
| **Rituximab** | 19 (26.0) | 14 (19.2) |
| **Mycophenolate** | 9 (12.3) | 9 (12.3) |
| **Azathioprine** | 5 (6.8) | 3 (4.1) |
| **Methotrexate** | 2 (2.7) | 1 (1.4) |
| **Cyclosporine** | 1 (1.4) | 0 |
| **Combination therapy*** | 11 (15.1) | 11 (15.1) |
| **Bilateral lung transplantation** | 1 (1.4) | 1 (1.4) |
| *Rituximab and steroids or azathioprine or mycophenolate | | |

**SUPPLEMENTARY TABLE 5. Multivariate analysis with CD21lo B cells considered as categorical variable (cut-off 10%)**

| **GLILD vs Controls**  **(n = 23 vs 66)** | **Odds Ratio**  **(95% C.I.)** | **P value** |
| --- | --- | --- |
| Splenomegaly | 7.79 (1.07-56.70) | 0.043 |
| CD21lo ≥ 10% | 31.42 (3.26-302.70) | 0.003 |
| DLCO (% of predicted) | 0.92 (0.87-0.98) | 0.011 |
| AI Cytopenia (ITP, AIHA) | 57.98 (3.81-881.47) | 0.003 |

***Logistic model for GLILD. Number of observations 89. Area under ROC curve = 0.97.***
